# The Stability of Oral Language Profiles of Children in the Early Years of School: A Longitudinal Comparison of Multidimensional and Cut‐Point Approaches to Classification

**DOI:** 10.1111/1460-6984.70246

**Published:** 2026-04-12

**Authors:** Anna Louise Taylor, Suze Leitão, Sharon Smart, Robyn Wheldall, Mary Claessen, Elien Vanluydt, Mark E. Boyes

**Affiliations:** ^1^ School of Allied Health Faculty of Health Sciences Curtin University Perth Australia; ^2^ Curtin enAble Institute Faculty of Health Sciences Curtin University Perth Australia; ^3^ Speech Pathology Australia Melbourne Victoria Australia; ^4^ Research Centre for Education and the Labour Market Maastricht University Maastricht the Netherlands; ^5^ Curtin School of Population Health Faculty of Health Sciences Curtin University Perth Australia

## Abstract

**Background:**

A cut‐point approach to classifying children's language abilities uses a specific threshold to determine whether an individual falls into a particular group, such as children with ‘typically developing language’ or ‘language difficulties.’ This method has been frequently used in longitudinal research to track language during the early school years. Findings have suggested that language difficulties may persist, emerge or resolve during this time.

**Aims:**

This longitudinal study with stratified sampling investigated oral language profiles using a multidimensional assessment framework, comparing results across multidimensional and cut‐point approaches and exploring how language profiles relate to children's functioning in Year 1.

**Methods:**

We assessed 90 children across multiple dimensions of oral language at school entry and followed them up one year later. A statistical method of combining data sources to look for groups with common characteristics (latent profile analysis) was used to identify language profiles and transitions between them. To compare the results with a cut‐point approach, children were subsequently reclassified into two groups using a single cut‐point from an omnibus test of oral language. Profile‐related differences in early academic and psychosocial outcomes were compared using a Multivariate Analysis of Variance. Follow‐up analyses using McNemar's test examined whether differences in classifications from the two classification methods were statistically significant.

**Results:**

Three language trajectory profiles were identified using the multidimensional approach: stable average, stable low and improving. The cut‐point method identified these same profiles and a small declining profile. Notably, more children were classified in the stable low group using the multidimensional approach compared to the cut‐point method, and this difference was statistically significant. In Year 1, children classified into language profiles characterised by average or above‐average abilities exhibited significantly stronger early academic outcomes compared to those in profiles associated with language difficulties.

**Conclusions:**

The use of a multidimensional assessment may result in greater consistency of categorical classifications over time for students with language difficulties. Further research is needed to explore the potential clinical utility of this approach to support the accurate and early identification of students with language difficulties and disorders.

**WHAT THIS PAPER ADDS:**

*What is already known on the subject*

- Previous studies that used cut‐point methods on scores from omnibus or domain‐specific tests have shown that some children demonstrate improving or declining oral language trajectories throughout their years of school. However, these methods may not fully capture the complexity of language growth and change over time.

*What this paper adds to the existing knowledge*

- This study offers new evidence on how a multidimensional assessment approach affects the classification and stability of language trajectories from school entry to Year 1. While a multidimensional approach identifies a greater number of children with language difficulties, it also reveals greater stability in language profiles over time, particularly for children with more significant challenges.

*What are the potential and actual clinical implications of this work?*

- The findings reinforce the need to adopt multidimensional assessment practices in research and clinical settings. Importantly, the high stability of language profiles identified using this method may increase clinician confidence in accurately identifying language difficulties at school entry.

## 1 Introduction

Many children's oral language difficulties remain unidentified throughout their years of schooling, imposing significant costs on education and healthcare systems (Cronin et al. 2017). The identification of children with language difficulties before or at the time of school entry should therefore be a priority (Adlof and Hogan 2019). Early detection of children who meet the criteria for Developmental Language Disorder (DLD) is crucial, as this prevalent condition affects approximately 6%–8% of children (Norbury et al. 2016). Individuals with DLD often face substantial academic challenges, along with difficulties in social adjustment and mental health (Iverson and Williams 2026).

As a key factor in diagnosing DLD, persistence signifies that language difficulties have been present for some time or have recently emerged but are expected to continue (Bishop et al. 2017). However, predicting whether language difficulties will be on‐going is complicated by individual variability in language development in the toddler and pre‐school years. Children with DLD are therefore not usually identified until after the age of four, although in some cases the disorder may not be detected until the emergence of functional impact on areas of everyday life (Iverson and Williams 2026).

### 1.1 Stability of Oral Language Development

Stability in language development refers to the maintenance of an individual's rank within a group of peers matched by age or grade over time. In contrast, instability is marked by shifts in rank, indicating that language ability relative to peers has improved or declined (Bornstein et al. 2018). Longitudinal studies of oral language development during early childhood (birth to four years) have highlighted the potential for instability due to periods of rapid growth and change. For example, the Early Years in Victoria Study (ELVS; Reilly et al. 2018) tracked Australian children's language using a standardised parent survey at age two and an omnibus test of oral language at age four. Children were classified as having ‘low language’ at age four if they achieved scores that fell >1.25 SD below the test mean. Among those classified as ‘late‐talkers’ at age two, 70% demonstrated typical language abilities by age four, while 8% of children with typical early language later showed low language ability. These findings highlighted the fluid nature of early language development in the years prior to starting school.

Research has suggested greater stability in language skills following school entry. For example, Norbury et al. (2017) tracked children's abilities in the first three years of school on measures of oral language, non‐verbal IQ and psychosocial functioning. All children, including those with language disorders, were observed to make measurable gains in oral language after school entry, meaning their relative rankings remained remarkably stable. Similarly, Bornstein et al. (2018) tracked children's language abilities from infancy through adolescence using multiple data sources, finding that a single extracted language variable remained highly stable over time.

Despite the evidence suggesting the likelihood of stability, other longitudinal research indicates a degree of on‐going variability in language performance after school entry. For instance, Reilly et al. 2018 measured pathways of language development of children from four to seven years of age. Participants were categorised as having either typical or impaired language based their performance relative to a cut‐point of 1.25 SD below the mean on a composite score derived from an omnibus test. While most children (86%) exhibited a stable profile (typical = 76%; low = 10%), around 14% experienced a shift (6% improving; 8% declining).

Another large‐scale Australian study (Zubrick et al. 2015), examined oral language trajectories of children at multiple time points from ages four to 10 using a measure of receptive vocabulary. While a large proportion of children (69%) demonstrated stable performance, others followed an improving (8%), declining (10%), or fluctuating (12%) pattern of development. Notably, less than 1% of children in this cohort exhibited stable low performance, highlighting the continued possibility of variability in oral language from early to middle childhood.

Longitudinal research from the UK has also revealed on‐going variability in childhood language development. For example, Snowling et al. (2016) examined the language abilities of children at ages three, five and eight. Using a cut‐point of 1.0 SD below the mean on a composite score from an omnibus test, four trajectories of language abilities were identified: stable typical (66%) resolving (6%), stable low (19%), and emerging language difficulties (10%). The authors concluded that ‘children with late‐emerging language impairments are relatively common and are hard to detect in the preschool years’ (p. 1360).

### 1.2 Factors Contributing to Instability in Oral Language

Previous research has explored factors associated with stability and instability of oral language pathways in childhood. For example, McKean et al. (2017) reported that environmental factors (e.g., social disadvantage) were linked to improving language, while biological factors (e.g., low birth weight) were associated with decline. Research also suggests the potential for early intervention and educational support to lead to changes in language abilities. For instance, a recent study (Bruinsma et al. 2023) tracked the progress of 154 four‐ to six‐year‐old children with DLD enrolled in an educational program offering specialised language intervention. Children demonstrated significant improvements multiple areas of oral language, suggesting that ‘catching up on language is possible for children with DLD’ (p. 766).

Some researchers have also highlighted the contribution of methodological factors in research studies to observed patterns of instability. Specifically, they have highlighted the potential for misclassifications when relying on cut‐points from omnibus or domain specific tests (Calder et al. 2023; Eadie et al. 2014). When used to track language over time, test scores that fall near a selected cut‐point may be particularly susceptible to minor fluctuations caused by extraneous factors, such as motivation, well‐being, or testing environment. Without considering a test's confidence interval (i.e., the range within which a child's true score is likely to fall), shifts in scores around the cut‐point at follow‐up may lead to changes in language classification that do not necessarily reflect meaningful improvement or decline in abilities or language functioning (Eadie et al. 2014).

Research examining the stability of DLD has typically conceptualised stability from a psychometric perspective, focusing on the consistency of individuals’ rank relative to peers over time (e.g., Bornstein et al. 2018; Norbury et al. 2017). However, there is growing recognition that clinicians and researchers should look beyond test scores to consider changes in how language is used in real‐world activities (McCormack et al. 2024). From this perspective, stability extends beyond rank‐order consistency to encompass functioning across time and contexts. Accordingly, any changes in language skills should be interpreted alongside the extent to which they reflect meaningful improvements in real‐world language use and participation (Thomas‐Stonell et al. 2019).

### 1.3 Evidence‐Based Assessment for Oral Language

Almost a decade ago, the CATALISE consortium (Bishop et al. 2016), published a series of consensus statements for the identification of children requiring support with oral language. They cautioned against the use of cut‐scores, acknowledging ‘there is no clear cut‐off that distinguishes between language impairment (regardless of its cause) from the lower end of normal variation of language ability’ (p.11). Alternatively, the CATALISE group advocated for a multidimensional assessment approach that evaluates language across multiple domains and considers the impact of difficulties on social and academic functioning, and participation. This approach was proposed as a more appropriate method for identifying children who require intervention and support, including those with DLD (Bishop et al. 2016). Combining multiple data sources has been suggested to enhance the diagnostic accuracy of assessment for DLD in other investigations (Iverson and Williams 2026; Sansavini et al. 2021).

In the second CATALISE study, Bishop et al. 2017 acknowledged that DLD is a broad, heterogeneous category of children who may display unique patterns of abilities in domains of oral language. While raising concerns about the limited empirical support and validation of existing diagnostic categories, they acknowledged the substantial evidence indicating that some children with language disorders exhibit specific impairments in the linguistic components of oral language (e.g., phonology and morphosyntax). They also noted that measures targeting specific markers of linguistic difficulties (e.g., nonword repetition or tense marking) have proven useful for identifying children with DLD. However, they also emphasised that not all children with language disorders experience difficulties with these tasks. Some children show specific weaknesses in other areas of oral language, such as word finding, pragmatics, discourse, and verbal learning and memory. Given the range of possible difficulties experienced by children with language disorders, Bishop et al. (2016) suggested that assessment should encompass tasks that tax a range of language components (e.g., sentence imitation, narrative production).

### 1.4 Evaluation of Language Functioning

One of the key points highlighted by the CATALISE consortium (Bishop et al. 2016) was the importance of considering how language difficulties affect functioning and participation in everyday activities. Language functioning relates to how children use language in everyday contexts (e.g., within learning activities at school and in participating socially in situations at home or in the community). Evaluation of language functioning is important, as research indicates that underlying linguistic ability does not always reliably predict everyday language use (Singer et al. 2023).

The timing of when functional consequences become evident may vary across individuals, and DLD may remain undetected until challenges in academic or social settings emerge (Iverson and Williams 2026). Understanding how profiles of underlying language ability relate to early academic and psychosocial functioning is important for identifying children at risk, informing early intervention, and tailoring supports to promote optimal developmental outcomes. In our previous investigation (Taylor et al. 2025), significant differences in both academic and psychosocial functioning were observed between higher and lower oral language profiles, highlighting the impact of language proficiency on functioning from the very beginning of formal schooling. However, differences between the two lower language profiles were not significant, suggesting that children with varying degrees of linguistic deficits may exhibit similar functional challenges. It remains unclear whether these patterns would continue as children transition into Year 1, or whether differences become more pronounced as both academic and social demands increase.

### 1.5 Limitations of Previous Studies

One of the most notable limitations of previous longitudinal studies of language pathways throughout the early years of school (e.g., Reilly et al. 2018; Zubrick et al. 2015) is their reliance on single cut‐point scores to dichotomously classify participants into groups of children with and without language difficulties. In research, a variety of data sources can be combined in a latent profile analysis (LPA), which offers a more nuanced approach to classification compared to cut‐point approaches. The use of LPA is therefore likely to provide a more person‐centred and ecologically valid classification system (Spurk et al. 2020). As such, results may better reflect current clinical decision‐making guidelines for detecting children who are likely to have significant and persistent language difficulties (Bishop et al. 2017).

Previous studies have also focused on measuring vocabulary and grammar at the word and sentence levels, with little emphasis on discourse, which relates to the use of language beyond individual sentences in longer stretches of communication within a social context (Dipper and Pritchard 2017). Measures of discourse complement word and sentence level measures of vocabulary and grammar by capturing how children use language in extended communicative contexts. The lack of longitudinal studies that have included measures of discourse represents a significant gap, particularly given research suggesting that discourse is a distinct dimension of oral language at school entry (Massonnié et al. 2022), and a strong predictor of both academic and psychosocial functioning in the first year of school (Taylor et al. 2025).

Given that previous methods to assessment in longitudinal studies differ from the recommendations of CATALISE (Bishop et al. 2016), it is crucial to examine how cut‐point‐based methods compare with multidimensional approaches. Although these approaches are not directly symmetrical in terms of the aspects of language they capture, comparing the estimates produced by each method can reveal whether they yield similar or divergent classifications of language difficulties and their stability over time. Additionally, this analysis will provide insight into the potential risks of misclassification when relying on cut‐points in research and clinical practice.

### 1.6 Aims of the Study

This longitudinal study aims to: (1) examine the stability of language profiles in the first two years of school using a multidimensional assessment framework; (2) compare the stability of language profiles identified through multidimensional and cut‐point approaches; and (3) explore the relationships between language profiles and functioning in Year 1.

Based on findings from previous longitudinal studies, we expected to observe at least four language trajectories: stable average (average language at both time points), improving (low to average), and declining (average to low), and stable low, with most participants expected to fall into the stable average group. Given that our approach was closely aligned with current guidelines for accurately assessing language difficulties and disorders (Bishop et al. 2016), greater stability in language profiles was expected compared to studies that used a cut‐point classification method. Based upon our prior findings indicating that children with mild to moderate language difficulties can display significant challenges in life areas related to language (Taylor et al. 2025), we anticipated observing functional similarities among the groups of children belonging to the lower profiles of oral language ability.

## 2 Method

### 2.1 Overview of the Study

This longitudinal study with stratified sampling assessed the oral language, early academic and psychosocial functioning, and word reading skills of children at two points in their educational journey: Pre‐primary (baseline) and Year 1 (follow‐up). Approval was granted from the Curtin University Human Research Ethics Committee (HRE2021‐0290) and the Department of Education of Western Australia.

### 2.2 Recruitment

Children were recruited from mainstream public schools in Western Australia located in the metropolitan area of Perth. In this educational context children typically commence full time school around five years of age. The mandated English curriculum includes formal instruction in early literacy, which includes both phonological awareness and phonics (Australian Curriculum and Reporting Authority [ACARA] 2025). For the baseline study (Taylor et al. 2025), five schools were selected based on their Index of Community Socio‐Educational Advantage (ICSEA; ACARA 2024), which measures school‐level socio‐economic background. Schools from each quartile were included in the sample. Of the five schools that took part of baseline, four accepted an offer to participate in this follow‐up study. There was a spread across all ICSEA quartiles in these schools, with most children (65%) attending schools in the middle two quartiles, and smaller proportions in the top (19%) and bottom (16%) quartiles. At these four schools, all families that participated previously in the study (*n* = 115) were invited to participate, with 78% (*n* = 90) accepting the offer. Attrition was primarily due to relocation or opting out. The majority of assessments (94%) were completed in Term 2.

### 2.3 Participants

The longitudinal sample included 90 students (47% male, 53% female), with average ages of 5 years and 4 months at baseline, and 6 years and 5 months at follow‐up. Children were eligible at baseline if English was their primary language and they had no diagnosed developmental or biomedical conditions that could affect language development. Parents of 7% of participants reported that their children were learning a language in addition to English at home. School Principals supported the identification of eligible participants by ensuring that information and consent forms were not sent home to families of children who had indicated on their enrolment forms that they were learning English as a second language, or who had a known diagnosis of a developmental disorder or medical condition affecting language. Follow‐up included all baseline participants, with no additional exclusion criteria. At baseline, 90% of parents completed a researcher‐developed questionnaire, capturing information on language background, family history of speech, language, and/or learning difficulties, and parental education. At follow‐up, a second questionnaire was completed by 92% of parents to capture information regarding access to speech pathology services. Of the children included in the study, a total of 16% were reported by their parents to have accessed speech pathology services between Pre‐primary and Year 1. Participant demographics are summarised in Table 1.

**TABLE 1:** Participants’ demographic and background information.

Variable	*n*	%	Variable	*N*	%
*Age at time of testing (years, months)*			*Family history of difficulties* ^*^		
5;6–5;11	6	6.7	Yes	19	21.1
6;0–6;5	43	47.8	No	59	65.6
6;6–6;11	41	45.6	Unsure	5	5.6
			Not reported	7	7.8
*Maternal education*			*Paternal education*		
Lower secondary (Year 10)	6	6.7	Lower secondary (Year 10)	7	7.8
Upper secondary (Year 12)	3	3.3	Upper secondary (Year 12)	6	6.7
Vocational education	18	20.0	Vocational education	30	33.3
Tertiary or advanced tertiary	55	61.1	Tertiary or advanced tertiary	40	44.4
Not reported	8	8.9	Not reported	7	7.8
*Language background*			*Service access* ^**^		
English only	78	86.7	No services accessed	65	72.2
English and other language(s)	6	6.7	Accessed services	16	17.8
Not reported	6	6.7	Not reported	9	10.0

*Note*: Percentages may not total 100% due to rounding.

*Speech, language, and/or learning difficulties.

**Access to speech‐language pathology services between baseline testing in Pre‐primary and follow‐up in Year 1.

### 2.4 Data Sources

Assessments investigated key dimensions of language (vocabulary, grammar, discourse) and functioning (early academic, psychosocial, word reading) using the same measures at both time points where possible to ensure a consistent approach.

#### 2.4.1 Vocabulary and Grammar

Four subtests (Sentence Comprehension, Word Structure, Expressive Vocabulary, Recalling Sentences) of the Clinical Evaluation of Language Fundamentals Preschool—third edition (CELF‐P3; Wiig et al. 2020) assessed skills in vocabulary and grammar.

#### 2.4.2 Discourse

Discourse production skills at baseline were assessed using the Squirrel Story Narrative Assessment (SSNA; Carey et al. 2006) and at follow‐up using the Peter and the Cat Narrative Assessment (PATC; Leitão and Allan 2003). The associated Narrative Comprehension Assessments (NCA; Dawes et al. 2018a, 2018b) assessed two forms of discourse comprehension: literal comprehension (understanding of information explicitly stated in the story) and inferential comprehension (children's ability to make inferences about the story). The SSNA and PATC are not identical tests but assess the same dimensions of oral narrative production (story organisation, story content, vocabulary, cohesion, use of referencing) and follow a comparable task format. The SSNA is best suited for children in Pre‐primary and PATC is aimed towards children in Year 1. Assessments were administered and scored using standard procedures. Despite their differences, the SSNA and PATC, along with their associated NCA assessments, were moderately to strongly positively correlated in this study (*r* = 0.544–0.630, *p* < 0.001), highlighting a meaningful degree of overlap between the tests.

#### 2.4.3 Communicative Participation

The Focus on the Outcomes of Communication Under Six (FOCUS‐34; Thomas‐Stonell et al. 2019) was used to capture children's communicative participation in daily interactions. The FOCUS‐34 is completed in two parts. In completing Part One, parents are asked to indicate on a seven‐point scale how well a statement describes their child (e.g., *My child uses correct grammar when speaking*). In Part Two, on the same scale, parents indicate how much help their child needs to complete an activity (e.g., *My child can be understood by other children*). Although the FOCUS‐34 is designed for children under six, given it is not a normed test and the relevance of items to the everyday lives of participants, it was deemed suitable to readminister at follow‐up (at which some participants were over age six). FOCUS‐34 scores demonstrated strong stability across the two time points (Spearman's ρ = 0.70, *p* < 0.001), indicating that children maintained relatively consistent rank ordering over time.

### 2.5 Early Academic Functioning

Early academic functioning in both Pre‐primary and Year 1 was captured using the Student Language Scale (SLS; Nelson et al. 2018), a measure of academic performance in classroom tasks related to oral language. On a scale of 1 (Not good) to 5 (Very good), teachers rated each participant's performance 12 items (e.g., *Understanding school vocabulary words; Figuring out new words when reading*). Scores for all test items were summed to calculate a total raw score.

#### 2.5.1 Psychosocial Functioning

To examine psychosocial functioning in an educational setting, the Australian version of the Strengths and Difficulties Questionnaire (SDQ; Goodman 2005) was completed by teachers at baseline and follow‐up. In completing the SDQ, teachers were asked to rate 25 statements on a 3‐point scale, based on their everyday observations of children's ability to manage emotions, sustain attention, regulate behaviour, and interact socially with peers. To facilitate comparison with other measures, reverse scoring was applied, so that higher scores indicated stronger psychosocial ability.

#### 2.5.2 Word Reading

The Test of Word Reading Efficiency—second edition (TOWRE‐2; Torgesen et al. 2012) was administered during the follow‐up phase. The Sight Word Efficiency (SWE) subtest measures the number of words (e.g., we, book) read in 45 seconds. The Phonemic Decoding Efficiency (PDE) subtest measures the number of nonsense words (e.g., wum, chur) accurately decoded in 45 seconds. The indices from both subtests were used to calculate the Total Word Reading Efficiency Index.

### 2.6 Statistical Methods

A speech pathologist double‐scored 20% of assessments, with high consensus (96% baseline, 92% follow‐up). Discrepancies were discussed until agreement was reached. Data were either complete or missing for 2% or less of the sample for all measures, except for the FOCUS‐34 (11% baseline, 7% follow‐up). A test of missing values (Little 1998) was conducted, revealing the FOCUS‐34 data were missing completely at random (baseline: *p* = 0.125; follow‐up: *p* = 0.141). Expectation Maximisation was applied in SPSS to impute estimations for missing values. To allow for comparison across the different measures, data were converted to Z‐scores.

#### 2.6.1 Stability of Language Profiles

While the sample size (*n* = 90) limited the complexity of statistical models that could be selected to examine the stability of language profiles, a LPA was considered appropriate in this context, as identification of profiles is influenced not only by sample size, but also by factors such as model specification and the degree of class separation (Tein et al. 2013). A single LPA was conducted in R Studio (version 12.1) using the tidyLPA package (Rosenberg et al. 2018). Data were structured in long format, with each participant represented twice (once for baseline and once for follow‐up). This allowed for simultaneous modelling of language profiles across time points. This approach to LPA has been used in other high‐quality published longitudinal research (e.g., Demaray et al. 2021; Vanluydt et al. 2022). All measures of oral language, including the FOCUS‐34, were included in the analysis. The selected model assumed equal variances across profiles, with covariances fixed to zero. Solutions with one to 12 profiles were explored in the initial analysis. To determine the number of profiles best fitting the data, a range of multiple fit indices were compared, including the Akaike Information Criterion (AIC), Bayesian Information Criterion (BIC), and sample‐size adjusted BIC (SABIC). The parsimony of the final model was then discussed with reference to theoretical and practical considerations. Profile memberships from the LPA were imported into SPSS for further analysis.

In consideration of the number of time points and sample size, a descriptive analysis was deemed the most appropriate method of examining changes in language profiles over time. Cross‐tabulations were therefore used to examine the patterns of stability by calculating the proportion of participants who remained in the same profile at follow‐up versus those who transitioned to a different profile.

#### 2.6.2 Comparison of Stability Estimates

Our second aim was to compare stability estimates derived from the multidimensional method with those from a cut‐point approach to classification. To implement the cut‐point approach, a variable was created based on children's performance on the CELF‐P3 Core Language Score (CLS; Wiig et al. 2020). Participants scoring at or below the cut‐point of 81 (1.25 SD below the test CLS mean) were classified as having language difficulties. This threshold is widely used to identify children at risk for language difficulties and has been applied in several epidemiological studies (e.g., Reilly et al. 2018; Tomblin et al. 1997), supporting comparison of results with other studies. Follow‐up analyses using McNemar's test were used to determine whether the proportion of children identified in each trajectory differed significantly between the multidimensional and cut‐point methods. This test was selected as it is appropriate for comparing paired categorical data from the same sample.

#### 2.6.3 Profile Related Differences in Functioning

A Multivariate Analysis of Variance (MANOVA) was conducted to examine relationships among oral language profiles and functioning. Total scores from the SLS (Nelson et al. 2018), TOWRE‐2 (Torgesen et al. 2012), and SDQ (Goodman 2005) were included as dependent variables, with profile membership as the categorical predictor. Follow‐up univariate analyses with a Bonferroni correction to control for Type 1 error were performed to explore differences in early academic functioning, psychosocial functioning, and word reading separately. Due to the relatively small size of the Very Low profile, which limited statistical power and the robustness of group comparisons, effect sizes and descriptive patterns were also considered when interpreting the results.

## 3 Results

### 3.1 Preliminary Analyses

An independent samples t‐test was conducted to compare baseline variables between the participants lost and retained at follow‐up. No significant differences were found in baseline the CELF‐P3 Core Language Score (*p* = 0.074), SDQ total score (*p* = 0.666), FOCUS‐34 total score (*p* = 0.507), or SLS total score (*p* = 0.496). Therefore, it was assumed the retained sample remained representative of the original cohort. Descriptive statistics for oral language and reading measures for the entire sample at baseline and follow‐up are presented in the Supplementary Materials (Table S1). Cohort scores on the CELF‐P3 (Wiig et al. 2020) and TOWRE‐2 (Torgesen et al. 2012) were consistent with published norms.

### 3.2 Profiles of Oral Language

**FIGURE 1:**
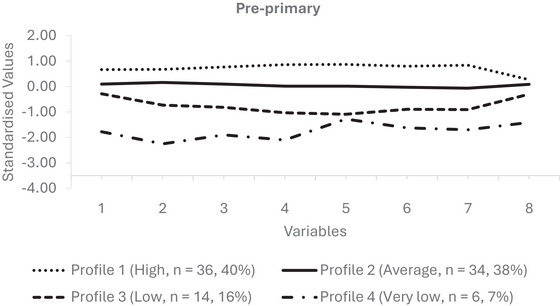
Profiles of Oral Language in Pre‐primary. Line graphs show the performance of oral language profiles across multiple measures in Pre‐primary, with profile means plotted as z‐scores relative to the cohort. *Note*: 1 = CELF‐P3 Sentence Comprehension; 2 = CELF‐P3 Word Structure; 3 = CELF‐P3 Expressive Vocabulary; 4 = CELF‐P3 Recalling Sentences (Wiig et al. 2020); 5 = NCA Literal 6 = NCA Inferential (Dawes et al. 2018a); 7 = SSNA (Carey et al. 2006); 8 = FOCUS‐34 (Thomas‐Stonell et al. 2019).

**FIGURE 2:**
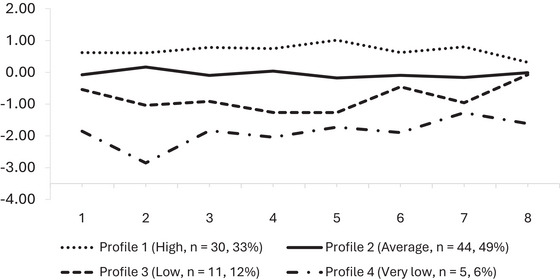
Profiles of Oral Language in Year 1. Line graphs show the performance of oral language profiles across multiple measures in Year 1, with profile means plotted as z‐scores relative to the cohort. *Note*: 1 = CELF‐P3 Sentence Comprehension; 2 = CELF‐P3 Word Structure; 3 = CELF‐P3 Expressive Vocabulary; 4 = CELF‐P3 Recalling Sentences (Wiig et al. 2020); 5 = NCA Literal; 6 = NCA Inferential (Daws et al. 2018b); 7 = PATC (Leitão and Allan 2003); 8 = FOCUS‐34 (Thomas‐Stonell et al. 2019).

Models meeting the minimum size criterion (>5% per profile; Table 2) were compared to identify the most accurate representation of oral language abilities. Although the AIC and SABIC values were similar for the four‐ and five‐profile solutions, the BIC favoured the four‐profile model, which also demonstrated strong classification accuracy (entropy = 0.87). Results from the Bootstrapped Likelihood Ratio Test (BLRT) were consistent across the two to four profile models (*p* = 0.01), with no significant improvement for a fifth profile (*p* = 0.08). Accordingly, the four‐profile solution was selected as the most parsimonious and interpretable representation of variation in oral language abilities. Profiles at Pre‐primary and Year 1 are shown in Figures 1 and 2, and corresponding mean scores and demographic variables for each profile are summarised in Table 3. The profiles represent heuristic groupings based on patterns in the data rather than definitive latent categories, providing a practical framework for identifying groups pf children with similar language abilities at both points in time.

**TABLE 2:** Fit indices for latent profile analysis.

Number of profiles	AIC	BIC	SABIC	Entropy	n_min	*p*BLRT
2	3598	3678	3599	0.95	0.21	0.01
3	3421	3529	3421	0.88	0.12	0.01
4	3369	3506	3370	0.87	0.06	0.01
5	3363	3530	3365	0.86	0.06	0.08

*Note*: Fit indices for latent profile models with two to five profiles. Model fit improved from the two to four profile solution, with lower AIC, BIC, and SABIC values, and high entropy across all models. The four‐profile model was selected as optimal based on fit, interpretability, and a significant bootstrapped likelihood ratio test.

Abbreviations: AIC, Akaike information criteria; BIC, Bayesian information criteria; n_min, smallest sample size across latent profiles; pBLRT, p‐value, Bootstrapped Likelihood Ratio Test; SABIC, sample‐size‐adjusted BIC.

**TABLE 3:** Oral language and reading mean scores (standard deviations) and demographic variables of oral language profiles in the early years of school.

	Pre‐primary (baseline)				Year 1 (follow‐up)			
	High	Average	Low	Very low	High	Average	Low	Very low
**Language variables**								
*CELF‐P3*								
Sentence comp	11.3 (2.3)	9.5 (1.9)	8.7 (2.1)	4.3 (2.3)	11.5 (2.0)	9.6 (1.9)	8.7 (2.7)	6.4 (1.1)
Word structure	11.3 (2.3)	9.4 (1.7)	6.9 (1.3)	4.0 (1.3)	13.1 (2.6)	10.6 (2.1)	7.5 (1.0)	5.4 (1.1)
Expressive vocabulary	11.7 (1.9)	9.9 (2.5)	6.9 (2.4)	4.0 (1.4)	13.0 (3.1)	9.3 (2.4)	7.0 (1.7)	5.8 (1.5)
Recalling sentences	12.1 (2.1)	9.3 (2.3)	6.4 (1.3)	3.8 (1.2)	13.4 (2.4)	10.2 (2.5)	6.8 (2.3)	5.6 (1.7)
CELF‐P3 CLS	108.4 (9.0)	96.6 (9.8)	83.4 (7.4)	69.2 (4.0)	115.7 (11.6)	98.4 (8.1)	85.7 (5.2)	76.4 (4.0)
*NCA*								
Literal	5.8 (1.0)	4.4 (0.8)	2.7 (1.3)	2.4 (1.4)	8.1 (1.5)	6.7 (1.6)	6.0 (1.0)	3.2 (2.6)
Inferential	14.2 (3.0)	10.7 (3.0)	7.1 (2.2)	4.0 (3.3)	16.0 (2.1)	11.9 (1.3)	8.2 (2.0)	6.6 (3.2)
Story retell^*^	13.9 (2.1)	11.1 (2.2)	8.4 (2.7)	5.9 (2.4)	14.0 (2.3)	10.5 (3.2)	7.6 (2.2)	6.5 (0.8)
FOCUS‐34	204.4 (28.1)	199.3 (25.1)	188.3 (22.1)	156.8 (49.1)	203.0 (18.4)	195.1 (19.6)	193.7 (14.2)	159.8 (42.3)
*TOWRE‐2*								
Phonemic decoding					107.6 (14.1)	96.9 (13.2)	88.5 (12.9)	91.8 (17.1)
Sight word					105.0 (19.2)	92.3 (14.9)	80.6 (12.1)	84.1 (12.0)
Total word reading					106.7 (16.9)	94.3 (14.0)	83.9 (11.1)	87.4 (14.7)
**Demographic variables**								
Child age in months	66.0 (2.8)	62.7 (3.6)	63.5 (3.7)	63.5 (3.7)	78.4 (3.1)	76.4 (3.7)	76.2 (4.2)	74.6 (3.9)
*Sex*								
Boys	44%	50%	57%	50%	63%	43%	73%	40%
Girls	56%	50%	43%	50%	37%	57%	27%	60%
*IRSAD quintile*								
Quintiles 1–2	0%	6%	33%	67%	0%	10%	36%	80%
Quintile 3	28%	18%	36%	0%	23%	23%	36%	0%
Quintile 4–5	73%	77%	21%	33%	77%	69%	27%	20%

*Note*: CELF‐P3 subtest scores are reported as standard scores (mean = 10, average range = 7–13). The CELF‐P3 Core Language Score and TOWRE‐2 scores are reported as index scores with a mean of 100 and SD of 15. All other scores are reported as raw scores.

Abbreviations: CELF‐P3, Clinical Evaluation of Language Fundamentals Preschool‐3 (Wiig et al. 2020; Dawes et al. 2018b); FOCUS‐34, Focus on the Outcomes of Communication Under Six (Thomas‐Stonell et al. 2019). TOWRE‐2, Test of Early Word Reading Efficiency second edition (Torgesen et al. 2012). IRSAD, Index of Relative Socio‐economic Advantage and Disadvantage, a measure of socio‐economic conditions ranked from least advantaged (Quintile 1) to most advantaged (Quintile 5); NCA, Narrative Comprehension Assessment (Dawes et al. 2018a).

*The Squirrel Story Narrative Assessment (Carey et al. 2006) was used in Pre‐primary. Peter and the Cat Narrative Assessment (Leitão and Allan 2003) was used in Year 1.

#### 3.2.1 Pre‐Primary (Baseline) Profiles

Profile 1 (High; *n* = 36; 40%) included children with the strongest oral language abilities relative to their peers, with group scores exceeding 0.5 SD above the cohort mean across all measures. Profile 2 (Average; *n* = 34; 38%) was characterised by oral language abilities consistently close to the cohort average. Profile 3 (Low; *n* = 14; 16%) showed a pattern of below average performance, with most scores up to 1.0 SD below the mean. Notably, scores on sentence imitation and literal comprehension for this group fell more than 1.0 SD below the mean. Profile 4 (Very Low; *n* = 6; 7%) represented the lowest‐performing group, with scores on receptive grammar, expressive vocabulary, literal and inferential comprehension, discourse production and communicative participation falling more than 1.0 SD below the mean. Expressive grammar scores for this group fell up to 2.3 SD below the cohort mean.

#### 3.2.2 Year 1 (Follow‐Up) Profiles

In Year 1, the distribution of language profiles closely mirrored those observed in Pre‐primary, with some variation in membership and mean oral language scores. Profile 1 (High; *n* = 30; 33%) showed reduced membership but demonstrated higher mean scores across all variables compared to Pre‐primary. Profile 2 (Average; *n* = 44; 49%) became the largest group, with all scores continuing to cluster around the cohort mean. Profile 3 (Low; *n* = 11, 12%) displayed a greater degree of difficulty in Year 1, with mean scores falling further below the cohort average than in Pre‐primary. Scores on expressive grammar and literal comprehension for Profile 3 fell up to 1.3 SD below the mean, although performance on the communicative participation measure remained close to average (0.09 SD below the cohort mean). Profile 4 (Very Low, *n* = 5; 6%) continued to show the most pronounced difficulties, with all scores falling more than1.5 SD below the mean, except for discourse production which fell 1.3 SD below. Expressive grammar emerged as a relative area of weakness for the Very Low group, with scores on measures of expressive morphology and sentence imitation falling up to 2.9 SD below the cohort mean.

### 3.3 Stability of Language Profiles

Profile memberships between Pre‐primary and Year 1 were cross tabulated to illustrate the movement between groups (see Table 4). Most participants in the High group in Pre‐primary (26/36; 72%) remained there in Year 1. All ten participants who shifted from this group continued to demonstrate language skills within the typical range. Strong stability was also observed in the Average group, with most participants (*n* = 30/34; 88%) remaining in that same group. Four participants from this group moved into the High group. For the Low group, 10/14 participants (71%) remained in their profile, but four (29%) moved up into the Average group. No participants from this group declined to a Very Low profile of oral language. The Very Low profile showed a high level of stability, with most participants (5/6, 83%) remaining in this group. One participant shifted out of the Very Low group into the Low group yet continued to present with language difficulties of a lesser severity. As such, this movement from the Very Low to Low group may be indicative of clinically relevant progress. However, the parent of this child reported that they did not access speech pathology services between Pre‐primary and Year 1, suggesting that factors other than oral language intervention delivered in a clinical context may have contributed to the shift for this individual.

**TABLE 4:** Cross tabulation of profiles at Pre‐primary and Year 1.

	Profile	Pre‐primary				
		High	Average	Low	Very Low	Total
Year 1	High	26	10	0	0	36
	Average	4	30	0	0	34
	Low	0	4	10	0	14
	Very low	0	0	1	5	6
	Total	30	44	11	5	90

*Note*: Cross‐tabulation of children's oral language profile classifications at Pre‐primary and Year 1. The table shows the number of children who remained in the same profile or transitioned between profiles across the two time points.

In examining the overall stability of the cohort from baseline to follow‐up, we considered both the potential influence of regression to the mean and the functional similarity of children across profiles (Taylor et al. 2025). At baseline, children in the Very Low and Low profiles exhibited broadly similar challenges in academic and psychosocial functioning, despite differences in language test scores. Consequently, a profile change was considered meaningful only when a child moved into or out of a profile characterised by average language abilities (i.e., from the Very Low or Low profile to the Average or High profile, or vice versa). This approach reflected a broader conceptualisation of stability and change, acknowledging that meaningful improvements or declines in language skills should be accompanied by corresponding changes in language performance and use. Based on this criterion, only four children (4% of the cohort) demonstrated a meaningful change, all moving from the Low profile up to the Average profile. The four children who showed improving profiles (three males and one female) were attending three different schools in three different ICSEA quartiles (low, middle‐upper, high). At school entry, all were reported by their parents to be monolingual speakers of English. Parents of three of these four children completed the parent questionnaire at follow‐up, and all reported that their child had not accessed speech pathology services between Pre‐primary and Year 1. The remaining 96% of the cohort maintained a stable classification over time (stable average: 78%, stable low: 18%).

### 3.4 Comparison of Methods

The second aim of this study was to compare the stability estimates identified using the multidimensional method with those derived from the cut‐point approach. Movement across the cut‐point (‐1.25 SD on the CELF‐P3 CLS; Wiig et al. 2020) was considered a change in classification. This approach identified a slightly higher proportion of children with stable, typical development (82%) compared to the multidimensional method (78%). The rates of children with an improving profile were also higher with the cut‐point approach (9%) than the multidimensional method (4%). Larger differences emerged in the classification of children within the lower profiles. Specifically, the stable low profile was less frequently identified using the cut‐point method (8%) compared to the multidimensional approach (18%). A small declining profile was only evident with the cut‐point method (1%). A summary of the comparison of stability estimates from both approaches is provided in Table 5.

**TABLE 5:** Comparison of stability estimates using multidimensional and cut‐point methods.

	Stable average	Improving	Declining	Stable low
Multidimensional	78%	4%	0%	18%
Cut‐point	82%	9%	1%	8%

*Note*: Estimates for the multidimensional approach reflect transitions into or out of a profile oral language profile of average abilities over time. Estimates for the cut‐point approach were derived by identifying children who moved above or below a threshold score set at 1.25 standard deviations below the mean on the Core Language Score of the CELF‐P3 (Wiig et al. 2020).

Follow‐up analyses using McNemar's test revealed no significant difference between the multidimensional and cut‐point approaches in identifying children with an improving trajectory (*p* = 0.344), and near‐identical classifications for the declining trajectory (*p* = 1.000). However, significant differences were found in the identification of children with stable average (*p* < 0.001) and stable low (*p* = 0.004) oral language trajectories. The full McNemar test results, including counts of concordant and discordant pairs of classifications, are presented in Table 6.

**TABLE 6:** Results of McNemar's Test comparing multidimensional and cut‐point classification methods across language trajectories.

Trajectory	Number of concordant pairs	Number of discordant pairs	Exact *p*‐value
Improving	80	10	0.344
Declining	89	1	1.000
**Stable average**	**71**	**19**	**<0.001**
**Stable low**	**81**	**9**	**0.004**

*Note*: Concordant pairs refer to the number of cases that were classified in the same trajectory category by both methods. Discordant pairs are the number of cases classified differently by the two methods. Significant results are bold.

### 3.5 Relationships Among Language Profiles and Functioning

**FIGURE 3:**
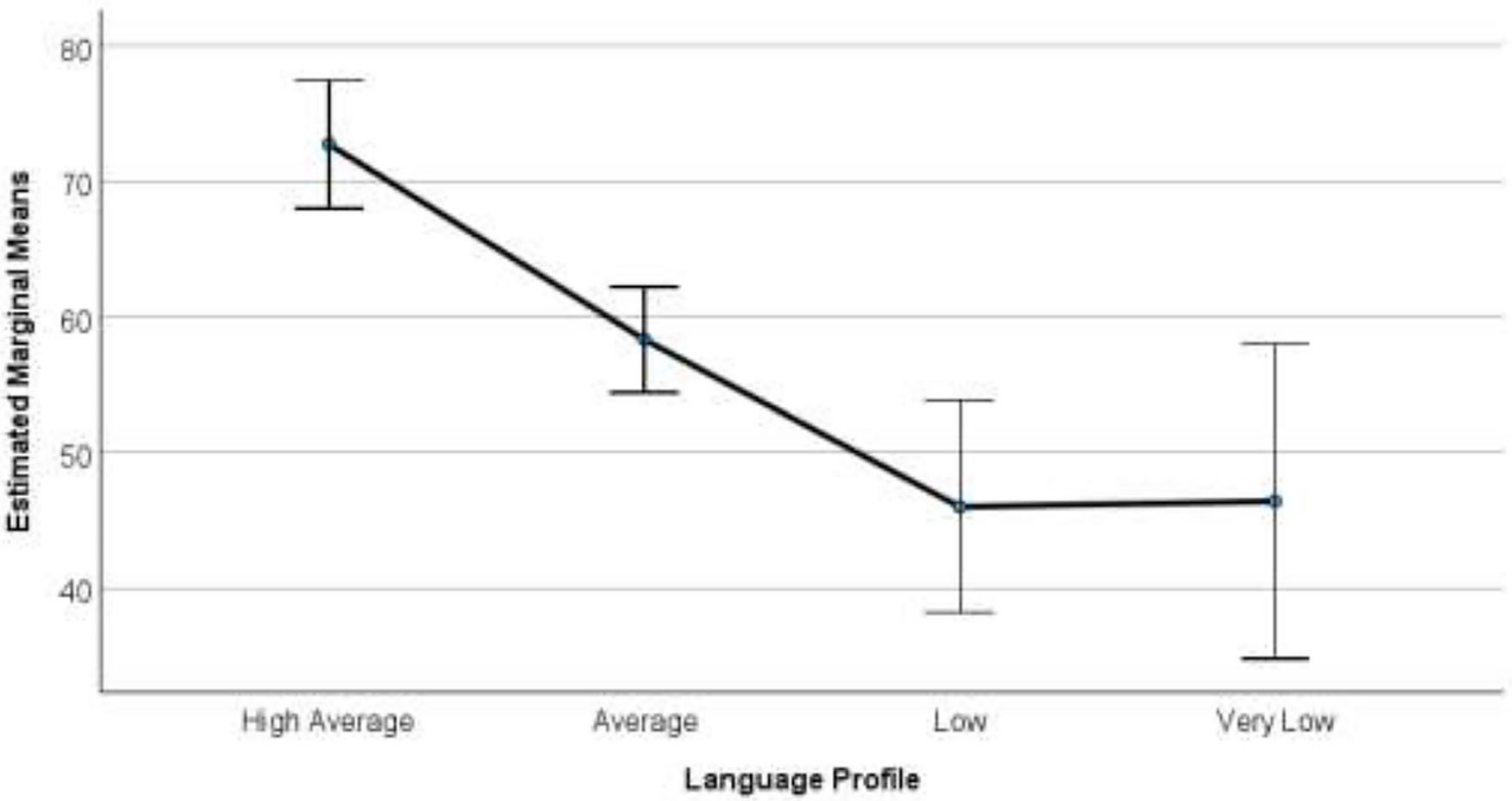
Estimated marginal means plots for early academic functioning in Year 1 across oral language profiles. Estimated marginal means plots with 95% confidence intervals show differences in early academic functioning across oral language profiles (error bars represent 95% confidence intervals).

**FIGURE 4:**
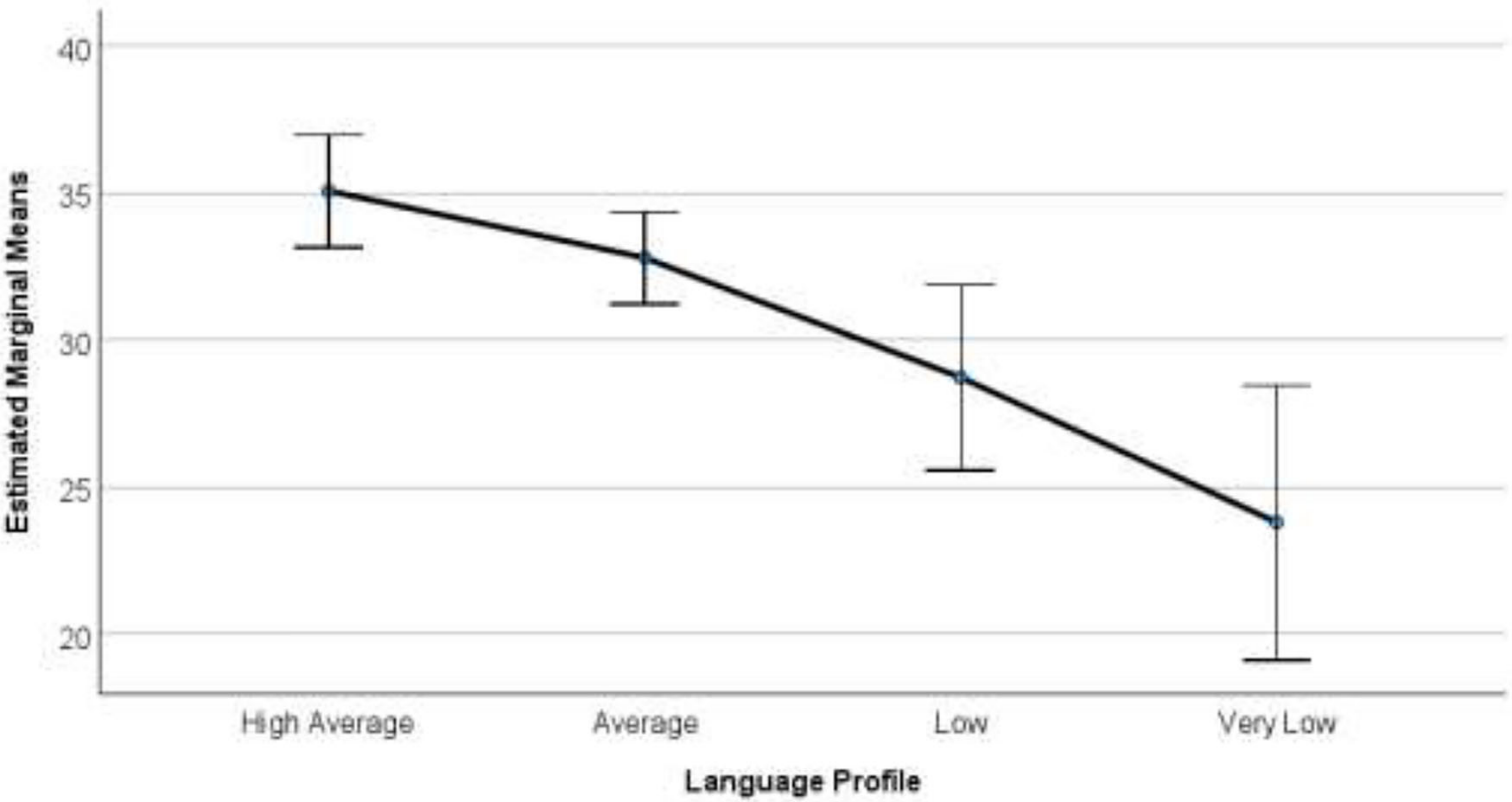
Estimated marginal means plots for psychosocial functioning in Year 1 across oral language profiles. Estimated marginal means plots with 95% confidence intervals show differences in psychosocial functioning across oral language profiles.

**FIGURE 5:**
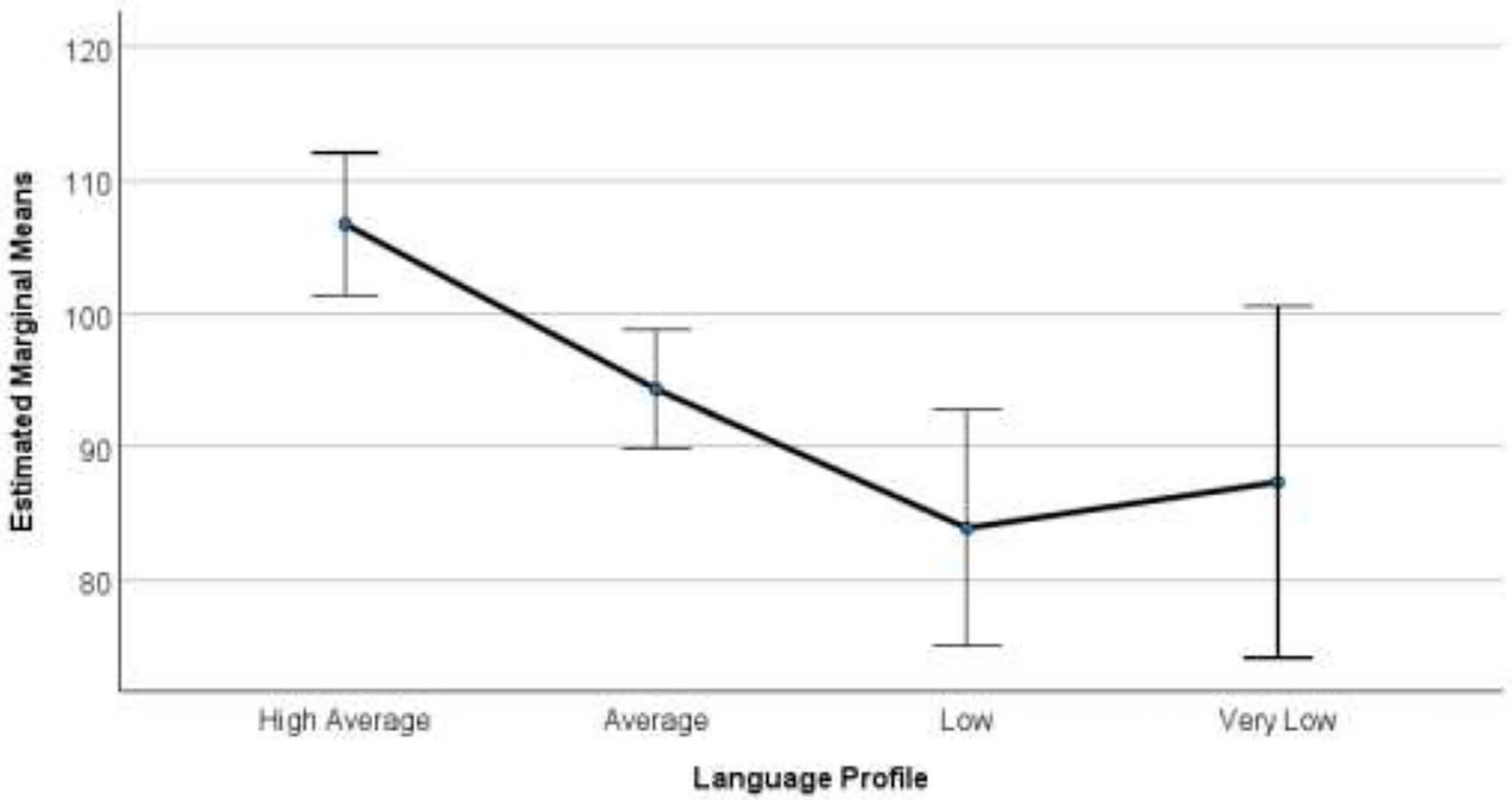
Estimated marginal means plots for word reading outcomes in Year 1 across oral language profiles. Estimated marginal means plots with 95% confidence intervals show differences in word reading across oral language profiles.

The third aim was to examine relationships among Year 1 oral language profiles in relation to functioning across life domains influenced by language. Results of the MANOVA indicated there was a statistically significant difference between profiles on the combined dependent variables, *F*(9, 205) = 5.24, *p* < 0.001, ηp^2^ = 0.16, Pillai's Trace = 0.46. Estimated marginal means plots by profile for each outcome variable are provided in Figures 3, 4 and 5. For early academic functioning, results revealed a significant main effect, *F(*3, 86) = 15.65, *p* = <0.001, ηp^2^ = 0.35. Significant differences were observed between all profiles (*p* < 0.05), except for the Very Low and Low groups. For word reading skills, there was also a significant main effect *F*(3, 86) = 8.35, *p* = <0.001, ηp^2^ = 0.23, and the High group was significantly different from all other groups (*p* < 0.05). However, no significant differences were observed among the remaining groups. For psychosocial functioning, there was also a significant main effect *F(*3, 86) = 8.87, *p* = <0.001, ηp^2^ = 0.24. Significant differences were observed between all profiles (*p* < 0.05), except the High and Average, Average and Low, and Low and Very Low groups. See Supplementary Materials (Tables S2, S3 and S4) for values relating to profile‐related differences across these domains.

## 4 Discussion

The first aim of this study was to examine the stability of oral language profiles of 90 children from Pre‐primary to Year 1 using a multidimensional assessment approach. Three language trajectories were identified: stable average (78%), stable low (18%), and improving (4%). The stable average group represented the largest proportion of the cohort (78%), closely aligning with Reilly et al.’s (2018) estimate of 76%, but exceeding rates reported by Snowling et al. (2016; 66%) and Zubrick et al. (2015; 69%). In contrast, 18% of children were classified in the stable low group, which is considerably higher than the proportions reported by Reilly et al. (2018; 10%) and Zubrick et al. (2015; <1%) but closely aligned with Snowling et al.’s (2016) estimate of 19%.

The proportion of children showing an improving trajectory (4%) was slightly lower than previously reported, which has ranged from 6% to 10% (Reilly et al. 2018; Snowling et al. 2016; Zubrick et al. 2015). All children who improved were initially classified in the Low profile and shifted to the adjacent Average profile at follow‐up. Descriptive analysis of the available parent survey data for three of the four children who showed an improving profile revealed that access to speech pathology services did not account for the observed gains in oral language. Furthermore, these children were attending schools across different areas of the social gradient, and their parents reported that only English was spoken in their homes. This suggests that environmental factors, such as language background and social disadvantage, are unlikely to explain their progress. These factors have been identified as contributors to improving language profiles in other studies (e.g., McKean et al. 2017). However, we acknowledge that the robustness of these observations is limited by the small number of participants in the improving group, and that we did not control for other potential factors such as school attendance, the presence of medical conditions, or other variables, which may have influenced their language development over the 12‐month period.

Notably, no children moved from the Very Low group to the Average or High profiles. This pattern suggests that, even without specialised intervention, children with language difficulties of a lower severity may be the most likely to make meaningful gains over time, compared to peers with more severe language difficulties. However, previous research has shown it is possible for some children with resolving language difficulties to experience re‐emergence as academic demands increase (Iverson and Williams 2026) highlighting the importance of on‐going monitoring for children with improving profiles in the early years of school.

The second aim of this study was to examine how classification methodology influences stability estimates by reclassifying children in the cohort using a cut‐point approach. Compared with the cut‐point method, the multidimensional approach identified a larger proportion of children with language difficulties at each time point. This aligns with our earlier findings (Taylor et al. 2025), which suggested that including discourse‐level and communicative participation measures may capture children with difficulties beyond word‐ and sentence‐level skills.

Longitudinally, children were more likely to change classification when the cut‐point approach was applied as it yielded higher rates of both improving (9% vs. 4%) and declining (1% vs. 0%) trajectories. In contrast, the multidimensional approach identified a significantly greater proportion of children in the stable low group (18% vs. 8%), suggesting it may be more sensitive to language difficulties that persist from the first to the second year of full‐time school.

Although overall proportions in the stable average trajectory were similar for both methods (multidimensional: 78%; cut‐point: 82%), the relatively large number of discordant pairs within this group (*n* = 19) highlighted some differences in individual classifications. Examination of these discordant cases showed that, under the multidimensional method, children were distributed across several trajectories: stable low (2/19), stable average (14/19), and improving (3/19). This pattern suggests that the multidimensional approach may provide a more differentiated classification of language profiles, potentially capturing more nunaced variation in patterns of language growth and change that the cut‐point method did not detect.

Interestingly, a declining profile was not observed using the multidimensional approach, and only one participant displayed this pattern using the cut‐point method. A possible explanation is that the comprehensive nature of the multidimensional method allowed for more accurate identification of language difficulties at the initial assessment, resulting in greater stability over time. Alternatively, the relatively short interval between assessments may not have been sufficient to detect a decline in abilities. Other longitudinal studies that identified declining trajectories (e.g., Snowling et al. 2016; Zubrick et al. 2015) had longer follow‐up periods, potentially with difficulties becoming more apparent as language demands increased. Another factor to consider is the role of biological risks (e.g., low birth weight,), which have been associated with declining language skills (McKean et al. 2017). The exclusion of children with developmental diagnoses and biomedical conditions in our sample may have reduced the likelihood of including those at higher biological risk.

In summary, compared with the cut‐point approach, the findings from this comparison of classification methodologies suggest that the multidimensional method may identify a higher prevalence of language difficulties while producing more stable categorical classifications of language ability. However, given the relatively small sample size for the aims of this investigation and the resulting constraints on the range of analytical approaches that could be applied, these findings should be interpreted with caution. It also remains possible that the cut‐point method is either more sensitive to true changes in language ability or more susceptible to spurious fluctuations. This may arise because the cut‐point approach relies on threshold‐based classification, which can detect small score changes that cross a cut‐off even when overall rank ordering remains similar. In contrast, the multidimensional approach integrates information across multiple domains and may therefore smooth over minor fluctuations, emphasising broader and more persistent patterns of language ability.

This work therefore opens an important line of inquiry to clarify the extent to which classification methodology influences prevalence and stability estimates of language difficulties. This may ultimately contribute to improving the diagnostic accuracy of language assessment at school entry, helping to ensure that children with on‐going language difficulties are identified from the commencement of formal schooling. As identification is often the first step toward intervention (Adlof and Hogan 2019), improved approaches to classification may facilitate earlier access to support and, in turn, better long‐term outcomes for children with language difficulties, including those with DLD.

The third aim was to explore the relationships between language profiles and academic and psychosocial functioning in Year 1. Effect sizes for profile differences in early academic skills, psychosocial functioning, and word reading were large (*ηp^2^* = 0.23–0.35), indicating that language profile membership accounted for a substantial proportion of variance in these outcomes. Children with stronger oral language profiles displayed stronger early academic abilities than those in the weaker groups. The Very Low group was not significantly different from the Low group, suggesting they may share similar functional challenges in academic tasks. These findings align with our initial investigation (Taylor et al. 2025), further solidifying our understanding of the relationship between language ability and early academic functioning for children with language difficulties.

Results were also similar for psychosocial functioning, where significant differences were observed among non‐adjacent language profiles (High and Low, High and Very Low, Average and Very Low). Groups displaying more similar language abilities (High and Average, Average and Low, Very Low and Low) appeared to demonstrate more similar social, emotional and behavioural skills.

Collectively, the findings on profile‐related differences in early academic and psychosocial functioning are consistent with previous research showing that children with mild language difficulties can experience challenges comparable to those with more severe difficulties (Eadie et al. 2014). These results underscore the importance of providing early support for children with language difficulties across the full continuum of severity. However, they should be interpreted with caution, as the relatively small size of the Very Low profile limited statistical power and the robustness of group comparisons, which may have contributed to the non‐significant pairwise comparisons.

A different pattern was observed for word reading, with only the High language profile showing significantly stronger skills than the other groups. This finding reinforces the view that strong oral language abilities support early progress in learning to read (Catts et al. 2012). However, the absence of significant differences between the Average, Low, and Very Low profiles in word reading skills may reflect limitations of the word‐level reading measure, which did not capture text‐level abilities or reading comprehension. Language difficulties may manifest in different ways as children grow older, evolving into challenges such as reading comprehension, and some children with resolving difficulties may also experience re‐emergence as academic demands increase (Iverson and Williams 2026).

### 4.1 Limitations

Generalisability of this study is likely limited by its relatively small sample size for the nature of the investigation and our sampling methods. Additionally, our sample only included children living in Perth, Western Australia with English as a primary language and may not be representative of larger or more diverse populations. Most participants were attending schools with an ICSEA value in the middle quartiles, and as such, the results may not be fully generalisable to communities with higher proportions of children from either disadvantaged or more advantaged backgrounds. Further, the short interval between test points may have influenced the observed patterns of growth and change. A larger, epidemiological study with a more diverse sample and a longer follow‐up period would provide more robust insights into the patterns of language growth and change. A larger study could also examine factors linked to improving language profiles, as the small number of children in this group limited analysis. Future research could look to explore the contribution of school‐related factors, such as the impact of educational programs, student attendance, and teacher training in supporting children with language difficulties.

There are also several other limitations that should be considered. While the comparison between the cut‐point and multidimensional approaches are informative, it should not be interpreted as a direct, like‐for‐like evaluation. Rather, the approaches differ in both the scope of constructs assessed and their underlying classification logic. As such, any observed differences may reflect not only variation in how individuals are classified, but also differences in the breadth of language abilities captured and the criteria used to assign individuals to groups.

While LPA offers many benefits and every effort was made to conduct it rigorously, there remains the possibility of spurious profiles emerging. This could occur if external factors, measurement errors, or unaccounted‐for variables influenced the identification of distinct profiles, leading to potentially misleading or oversimplified conclusions about the underlying data (Spurk et al. 2020). Furthermore, prior to conducting the MANOVA, we examined key assumptions. Tests of normality (Kolmogorov‐Smirnov and Shapiro‐Wilk) indicated deviations for SLS and SDQ, with TOWRE closer to normality. Levene's test suggested equality of variances for TOWRE and borderline results for SDQ, but a violation for SLS. Additionally, Mahalanobis distance analysis identified one multivariate outlier. While these findings indicate modest assumption violations, MANOVA is generally considered robust to such issues when sample sizes are adequate (Field 2009; Pallant 2020). Our smallest group included five participants, meeting the minimum recommended size for this analysis. Nevertheless, these limitations should be considered when interpreting the results, particularly given that MANOVA can be sensitive to attrition, which may have influenced the observed effects.

Regarding measurement‐related limitations, it is important to acknowledge that differences in test design between the SSNA and PATC may have contributed to some of the observed longitudinal variability. As such, changes in scores related to discourse‐level communication should be interpreted with this consideration in mind. Furthermore, the FOCUS‐34, originally designed for children under six, may have lacked the sensitivity to capture the broader and more complex communicative contexts that children encounter in Year 1. As children grow older, they are typically involved in more complex learning and social interactions (e.g., class discussions, partner work) and are expected to engage with a wider range of communication partners (Ninio and Snow 2018). These increased expectations in communicative participation may not have been fully captured by the FOCUS‐34, which was developed with a younger cohort in mind. Consequently, interpretations of functional stability based on this measure should be considered with caution. Additionally, the measures of academic and psychosocial functioning may not have been sensitive enough to distinguish between lower language profiles. Future research could also consider developing robust tools to capture language functioning in school‐aged children. Finally, it is important to note his study was not diagnostic in nature, and participants were not formally diagnosed with DLD. As such, exploring the diagnostic specificity of the multidimensional measure used in this study was beyond the scope of our investigation.

### 4.2 Clinical Implications

Although the findings of this work should be interpreted cautiously, they offer several conceptual considerations for speech pathologists who are seeking to assess and identify language difficulties at school entry. Clinicians who adopt a multidimensional assessment of language may be relatively confident that language difficulties identified at school entry will persist into Year 1. As statistical procedures to combine data sources are not typically used in clinical contexts, to support clinical reasoning and diagnostic decision‐making, speech pathologists may find it useful to map data onto the framework for the identification of language disorders as outlined by Bishop et al. (2016). To determine the *significance* of language difficulties, results from standardised tests of vocabulary, grammar and discourse may be used to determine performance relative to norms. To evaluate the *functional impact* of language difficulties, information from teachers pertaining to early academic and psychosocial functioning can be efficiently gathered using standardised surveys, such as those used in this study. Furthermore, a thorough case history can be examined to rule out the likelihood the language difficulties may be transient or attributed to another cause. When the *persistence* of language difficulties from early childhood to school entry is uncertain, clinicians may have greater confidence in knowing that if children are identified with language difficulties at school entry using a multidimensional method, these difficulties are more likely to persist than resolve in the first year of school. Additionally, as the findings suggest that children with mild to moderate language difficulties may also be at risk for academic and psychosocial challenges, clinicians may therefore wish to consider extending support beyond those with the most severe difficulties to promote language development and related functioning.

Further research is needed to validate and support the use multidimensional assessment methods and integration of data sources in clinical contexts. Given the time and expertise required to administer and interpret the assessments in a multidimensional approach, future research could explore which measures are the most cost‐effective and resource‐efficient, helping to refine and streamline the assessment battery. This could also contribute to the development of a multidimensional language assessment tool that teachers could use to identify children at risk and in need of further, more comprehensive evaluation by a speech pathologist.

## 5 Conclusion

In summary, this study suggests that classification methodology influences both the identification and stability of language difficulties in the early years of school. Compared with a cut‐point approach, a multidimensional method identified a larger proportion of children with language difficulties but yielded more consistent classifications over time. Language profile‐related differences in academic and psychosocial outcomes indicate that children with mild to moderate language difficulties may experience functional challenges comparable to those with more severe difficulties. These findings offer preliminary conceptual guidance for clinicians, indicating that multidimensional assessments may help identify children with language difficulties and related functional challenges who could otherwise go unrecognised. Findings from this study should be interpreted cautiously given the relatively small sample, measurement‐related limitations and the 12‐month follow‐up period. Future research could apply a multidimensional assessment approach to a larger sample with longer follow‐up to examine the potential for these methods to enhance early identification and support.

## Funding

The lead author expresses gratitude to the Curtin University Aboriginal and Torres Strait Islander Scholarship and the Bertrond and Edith Donohue Scholarship for financial support. Mark Boyes was supported by the National Health and Medical Research Council, Australia (Investigator Grant 1173043) and the Stan Perron Charitable Foundation (People Grant 202405).

## Ethics Statement

Ethical approval for this study was obtained from the Curtin University Human Research Ethics Committee (Approval Number: HRE2021‐0290), the Department of Education of Western Australia (DoE), and Catholic Education Western Australia (CEWA). Written informed consent was obtained from all parents/carers of participating children, as well as school principals and teachers. In addition, verbal assent was obtained from each child participant prior to testing.

## Conflicts of Interest

Anna Louise Taylor is employed by MultiLit Pty Ltd, an educational program provider that develops programs that aim to improve language and literacy skills in children. Robyn Wheldall is a director of MultiLit Pty Ltd and has a financial interest in it.

## Supporting information




**Supporting Table S1**: Descriptive statistics on oral language and reading measures. **Supporting Table S2**: Profile related differences in early academic functioning in Year 1. **Supporting Table S3**: Profile related differences in psychosocial functioning in Year 1. **Supporting Table S4**: Profile related differences in word reading skills in Year 1.

## Data Availability

Data that support the findings of this study are available from the corresponding author upon reasonable request.
