# Supplementary material for: The Stability of Oral Language Profiles of Children in the Early Years of School: A Longitudinal Comparison of Multidimensional and Cut‐Point Approaches to Classification
Source: Int J Lang Commun Disord. 2026 Apr 12;61:e70246. doi: 10.1111/1460-6984.70246 (PMC13071115; doi:10.1111/1460-6984.70246)
Supplement: Supplementary file 1 — Supporting Table S1: Descriptive statistics on oral language and reading measures. Supporting Table S2: Profile related differences in early academic functioning in Year 1. Supporting Table S3: Profile related differences in psychosocial functioning in Year 1. Supporting Table S4: Profile related differences in word reading skills in Year 1. [file JLCD-61-0-s001.docx]

**Supplementary Materials**

**Tables**

Table S1. Descriptive statistics on oral language and reading measures

Table S2. Profile related differences in early academic functioning in Year 1

Table S3. Profile related differences in psychosocial functioning in Year 1

Table S4. Profile related differences in word reading skills in Year 1

**TABLE S1. Descriptive statistics on oral language and reading measures**

|  | Pre-primary (baseline) | | Year 1 (follow-up) | | |
| --- | --- | --- | --- | --- | --- |
|  | Mean (SD) | Range | Mean (SD) | | Range |
| *CELF-P3* |  |  |  | |  |
| Sentence Comprehension | 9.6 (2.8) | 1–16 | 10.0 (2.4) | | 4–14 |
| Word Structure | 9.4 (2.8) | 3–16 | 10.8 (3.1) | | 4–16 |
| Expressive Vocabulary | 9.7 (3.1) | 2–16 | 10.1 (3.4) | | 4–18 |
| Recalling Sentences | 9.6 (3.4) | 2–19 | 10.6 (3.4) | | 3–17 |
| Core Language Score | 96.3 (14.5) | 66–128 | 101.4 (14.8) | | 70–141 |
| *NCA* |  |  |  | |  |
| Literal | 4.5 (1.6) | 0.5–9 | 6.8 (1.9) | | 0–11 |
| Inferential | 11.1 (4.3) | 0–21 | 12.5 (3.5) | | 1–20 |
| Story retell* | 11.5 (3.3) | 1.5–18 | 11.09 (3.6) | | 3.5–18 |
| FOCUS-34 | 196.8 (28.4) | 75–234 | 195.6 (22.15) | | 89–226 |
| *TOWRE-2* |  |  |  |  | |
| Phonemic Decoding Efficiency |  |  | 99.2 (15.0) | 60–142 | |
| Sight Word Efficiency |  |  | 94.6 (17.9) | 64–145 | |
| Total Word Reading Efficiency |  |  | 96.8 (16.5) | 60–146 | |

*The Squirrel Story Narrative Assessment (Carey et al., 2006) was used in Pre-primary. Peter and the Cat Narrative Assessment (Leitao & Allan, 2003) was used in Year 1.

*Abbreviations.* CELF-P3 = Clinical Evaluation of Language Fundamentals Preschool-3 (Wiig et al., 2020). NCA = Narrative Comprehension Assessment (Dawes et al., 2018a, Dawes et al., 2018b). FOCUS-34 = Focus on the Outcomes of Communication Under Six (Thomas-Stonell et al., 2015). TOWRE-2 = Test of Early Word Reading Efficiency second edition (Torgesen et al., 2012).

*Note.* CELF-P3 subtest scores are reported as standard scores (mean = 10, average range = 7–13). The CELF-P3 Core Language Score and TOWRE-2 scores are reported as index scores with a mean of 100 and SD of 15. All other scores are reported as raw scores.

**TABLE S2. Profile related differences in early academic functioning in Year 1**

| Dependent  variable | Language profile | Language profile | Mean difference (I-J) | Std. error | Sig. |
| --- | --- | --- | --- | --- | --- |
| SLS total score | High | Average | 14.4* | 3.1 | <0.001 |
|  |  | Low | 26.7* | 4.6 | <0.001 |
|  |  | Very Low | 26.3* | 6.3 | <0.001 |
|  | Average | High | -14.4* | 3.1 | <0.001 |
|  |  | Low | 12.3* | 4.4 | 0.038 |
|  |  | Very Low | 11.9* | 6.2 | 0.034 |
|  | Low | High | -26.7* | 4.6 | <0.001 |
|  |  | Average | -12.3* | 4.4 | 0.038 |
|  |  | Very Low | -0.4 | 7.0 | 1.000 |
|  | Very Low | High | -26.3* | 6.3 | <0.001 |
|  |  | Average | -11.9 | 6.2 | 0.034 |
|  |  | Low | 0.4 | 7.0 | 0.286 |

*The mean difference is significant at the 0.05 level.

*Note.* SLS = Student Language Scale (Nelson et al., 2018)

**TABLE S3. Profile related differences in psychosocial functioning in Year 1**

| Dependent  variable | Language profile | Language profile | Mean difference (I-J) | Std. error | Sig. |
| --- | --- | --- | --- | --- | --- |
| SDQ total score | High | Average | 2.3 | 1.3 | 0.430 |
|  |  | Low | 6.3* | 1.9 | 0.006 |
|  |  | Very Low | 11.3* | 2.5 | <0.001 |
|  | Average | High | -2.3 | 1.2 | 0.430 |
|  |  | Low | 4.1 | 1.8 | 0.145 |
|  |  | Very Low | 9.0* | 2.5 | 0.003 |
|  | Low | High | -6.4* | 1.9 | 0.006 |
|  |  | Average | -4.1 | 1.8 | 0.145 |
|  |  | Very Low | 4.9 | 2.8 | 0.516 |
|  | Very Low | High | -11.3* | 2.5 | <0.001 |
|  |  | Average | -9.0* | 2.5 | 0.003 |
|  |  | Low | -4.9 | 2.8 | 0.516 |

*The mean difference is significant at the 0.05 level.

*Note.* SDQ = Strengths and Difficulties Questionnaire (Goodman, 2005)

**Table S4.** *Profile related differences in word reading skills in Year 1*

| Dependent  variable | Language profile | Language profile | Mean difference (I-J) | Std. error | Sig. |
| --- | --- | --- | --- | --- | --- |
| TOWRE_2 total score | High | Average | 12.4* | 3.5 | 0.004 |
|  |  | Low | 22.8* | 5.2 | <0.001 |
|  |  | Very Low | 19.3* | 7.2 | 0.049 |
|  | Average | High | -12.4* | 3.5 | 0.004 |
|  |  | Low | 10.4 | 5.0 | 0.240 |
|  |  | Very Low | 7.0 | 7.0 | 1.000 |
|  | Low | High | -22.8* | 5.2 | <0.001 |
|  |  | Average | -10.4 | 5.0 | 0.240 |
|  |  | Very Low | -3.5 | 8.0 | 1.000 |
|  | Very Low | High | -19.4* | 7.2 | 0.049 |
|  |  | Average | -7.0 | 7.0 | 1.000 |
|  |  | Low | 3.4 | 8.0 | 1.000 |

*The mean difference is significant at the 0.05 level.

*Note.* TOWRE-2 = Test of Word Reading Efficiency second edition (Torgesen et al., 2012)
